# Structural bases for the higher adherence to ACE2 conferred by the SARS‐CoV‐2 spike Q498Y substitution

**DOI:** 10.1107/S2059798322007677

**Published:** 2022-08-25

**Authors:** Elena Erausquin, Fabian Glaser, Juan Fernández-Recio, Jacinto López-Sagaseta

**Affiliations:** ^1^ Navarrabiomed Unit of Protein Crystallography and Structural Immunology Pamplona 31008 Spain; ^2^ Public University of Navarra (UPNA) Pamplona 31008 Spain; ^3^ Navarra University Hospital Pamplona 31008 Spain; ^4^ Technion – Israel Institute of Technology The Lorry I. Lokey Center for Life Sciences and Engineering Haifa Israel; ^5^ CSIC–UR–Gobierno de La Rioja Instituto de Ciencias de la Vid y del Vino (ICVV) Logroño Spain

**Keywords:** SARS‐CoV‐2, COVID‐19, X‐ray structure, spike protein Q498Y mutation, binding affinity, spike protein receptor binding domain, RBD–ACE2 complex, ACE2 receptor

## Abstract

A remarkable number of SARS‐CoV‐2 variants and other as yet unmonitored lineages harbor amino‐acid substitutions with the potential to modulate the interface between the spike receptor‐binding domain (RBD) and its receptor ACE2. The naturally occurring Q498Y substitution, which is present in currently circulating SARS‐CoV‐2 variants, has drawn the attention of several investigations. While computational predictions and *in vitro* binding studies suggest that Q498Y increases the binding affinity of the spike protein for ACE2, experimental *in vivo* models of infection have shown that a triple mutant carrying the Q498Y replacement is fatal in mice. To accurately characterize the binding kinetics of the RBD Q498Y–ACE2 interaction, biolayer interferometry analyses were performed. A significant enhancement of the RBD–ACE2 binding affinity relative to a reference SARS‐CoV‐2 variant of concern carrying three simultaneous replacements was observed. In addition, the RBD Q498Y mutant bound to ACE2 was crystallized. Compared with the structure of its wild‐type counterpart, the RBD Q498Y–ACE2 complex reveals the conservation of major hydrogen‐bond interactions and a more populated, nonpolar set of contacts mediated by the bulky side chain of Tyr498 that collectively lead to this increase in binding affinity. In summary, these studies contribute to a deeper understanding of the impact of a relevant mutation present in currently circulating SARS‐CoV‐2 variants which might lead to stronger host–pathogen interactions.

## 1. Introduction

Coronavirus disease 2019 (COVID-19) is a currently persistent threat that is being counteracted by means of massive vaccination campaigns in humans. While vaccination routines are associated with a reduction in severity and with improved clinical outcomes, the recurrent emergence of novel severe acute respiratory syndrome coronavirus (SARS-CoV-2) variants poses new challenges for which there are currently no universal preventive measures. Since the first COVID-19 outbreak, a large number of SARS-CoV-2 variants have surfaced, some of which have triggered new pandemic episodes (Lazarevic *et al.*, 2021; Harvey *et al.*, 2021). Some of these variants are characterized by mutations that affect the receptor-binding domain (RBD) binding site on ACE2 (Supplementary Fig. S1). A variant bearing the N501Y mutation was detected in the United Kingdom, referred to as Variant of Concern (VOC) 202012/01 or the alpha variant, following the World Health Organization convention (https://www.who.int/en/activities/tracking-sars-cov-2-variants/), and was linked to increased transmissibility (Davies *et al.*, 2021).

The 501.V2 variant, or beta variant, was originally identified in South Africa in December 2020 (Tegally *et al.*, 2020). While the prevalence of this variant was higher among young people, it correlates with a more severe clinical condition. Further, this variant appears to disseminate at a higher rate compared with previously identified SARS-CoV-2 variants. SARS-CoV-2 beta has three amino-acid substitutions in the RBD. These are N501Y, K417N and E484K, which appear to enhance the binding strength of the spike protein to ACE2 (Dejnirattisai *et al.*, 2021). The delta variant became predominant in many countries and includes L452R and T478K substitutions in the RBD. The delta plus variant includes an extra amino-acid replacement, K417N (Kannan *et al.*, 2021). The most recent SARS-CoV-2 VOC emerged in the last quarter of 2021, and is referred to as omicron (B.1.1.529 and BA lineages). It is currently dominant and has spread worldwide, showing the fastest transmission rate of all SARS-CoV-2 variants to date. Moreover, the large number of mutations present in the spike protein (15 of which are found in the RBD) provides this variant with mechanisms of immune evasion. Indeed, it can infect previously immunized or infected individuals (Liu *et al.*, 2022).

At a molecular level, SARS-CoV-2 initiates contact with the host through its viral envelope-anchored trimeric spike protein, which binds with very high affinity to ACE2 on the surface of human cells (Walls *et al.*, 2020). Many structural studies have determined the structural and molecular blueprint of this binding in great detail, which involves an array of both polar and nonpolar interactions between the spike RBD and ACE2 (Shang *et al.*, 2020; Lan *et al.*, 2020; Zhou *et al.*, 2020). The binding interface covers a molecular area of 850 $Å^2$. 18 residues on the spike RBD establish direct interactions with ACE2 through bonds of varying nature, including hydrogen bonds, van der Waals forces and salt bridges. With the aim of mapping mutations in the spike RBD that could lead to enhanced adherence to ACE2, we performed *in silico* predictions and identified diverse individual substitutions that were predicted to increase the binding of wild-type RBD to ACE2 to varying degrees. Amongst these favorable substitutions, Q498Y showed the greatest impact. Interestingly, the Q498Y substitution has already drawn the attention of other groups, who have assessed its impact on the binding to ACE2 using varied approaches. These include *in silico* prediction and molecular-dynamics procedures (Yi *et al.*, 2020; Capponi *et al.*, 2021; Li *et al.*, 2020; Ahamad *et al.*, 2022), and yeast display and deep mutational scanning (Smyth *et al.*, 2022), which point to an enhanced affinity associated with the Q498Y substitution, while animal models have even shown that Q498Y combined with two other mutations in the spike leads to lethal infection (Iwata-Yoshikawa *et al.*, 2022). Further, very recent studies conceived to explore the emergence of novel variants have detected an increased number of SARS-CoV-2 lineages of unknown host which, apart from sharing a large number of the mutations found in omicron, included the Q498Y substitution (Smyth *et al.*, 2022). All in all, these results support the presence of a massive number of circulating SARS-CoV-2 variants that could ultimately lead to novel threats to humankind. Given these precedents, on the one hand, the widespread interest in the Q498Y substitution and, on the other hand, the continuous and unpredictable emergence of novel SARS-CoV-2 variants carrying mutations in the RBD–ACE2 binding interface, we set out to investigate the molecular and structural bases for the stronger adherence caused by this substitution, which remain elusive.

Here, we report the biophysical and structural characterization of the RBD Q498Y–ACE2 complex and describe the molecular and structural grounds underpinning its increased affinity.

## 2. Materials and methods

### 2.1. *In silico* mutational screening

We performed high-throughput *in silico* mutagenesis using structural information on the interface between RBD and ACE2. This information was collected from the atomic coordinates deposited in the Protein Data Bank as PDB entry 6m0j and was used to feed the *mCSM-PPI*2 pipeline (Rodrigues *et al.*, 2019). Residues located at the RBD–ACE2 interface were subjected to replacement by all possible natural amino acids. Thus, *mCSM-PPI*2 scans all possible mutations at every position, calculating for each replacement, individually, a prediction of the change in Gibbs free energy ($\Delta\Delta G$). The output is provided as a heatmap in which every position that has been mutated is assigned a color and intensity according to a scale-bar gradient that mirrors the impact of the single substitution on the overall binding affinity.

### 2.2. Cloning and generation of recombinant baculovirus

Codon-optimized gene sequences for human extracellular ACE2 containing a C-terminal 12×His tag and for SARS-CoV-2 wild-type spike RBD (with position 334 being the first N-terminal native amino acid) with an N-terminal TwinStrep tag followed by a human rhinovirus 3C protease site were synthesized by BioBasic Inc. The RBD beta variant RBD (RBD $\beta$) was synthesized by GeneUniversal, with position 334 being the first N-terminal native amino acid. Sequences were digested from generic delivery vectors using BamHI and NotI restriction enzymes (FastDigest) and were cloned in the pAcGP67A transfer vector using Optizyme T4 DNA Ligase (ThermoFisher). *Escherichia coli* DH5$\alpha$ cells (Invitrogen) were transformed with the modified transfer vectors before plasmid DNA extraction using a GeneJET Plasmid Miniprep Kit (ThermoFisher Scientific) following the manufacturer's instructions. An additional ACE2 construct was generated via PCR with an N-terminal TwinStrep tag and 3C site upstream of the protein sequence. N-terminal TwinStrep–3C site RBD constructs including the individual Q498Y (RBD Q498Y), K417N (RBD K417N), E484K (RBD E484K) and N501Y (RBD N501Y) substitutions and the triple Q493K/Q498Y/P499T mutant (RBD Q493K/Q498Y/P499T) were generated by PCR using specific primers. To enable protein isolation from the culture supernatant, all protein genes used in this study were cloned in frame and downstream of the gp67 secretion signal sequence included in the pAcGP67A

baculovirus transfer vector. All new PCR products were further digested and cloned into pAcGP67A vectors as described previously.

Once the sequences had been validated by Sanger sequencing, Sf9 insect cells (Gibco) were transduced with each transfer plasmid, BestBac 2.0 Δ v-cath/chiA Linearized Baculovirus DNA and Expres2 TR Transfection Reagent (Expression Systems) to produce the final recombinant baculovirus.

Initial recombinant baculoviruses were collected five days after transduction and incubation at 28°C. Virus titers were amplified by infecting fresh Sf9 cultures at a density of $1 \times 10^6$ cells $ml^{-1}$ in Sf-900 III SFM (ThermoFisher). Cell morphologies and densities were examined on a daily basis for infection. After 24–48 h, the cells had a swollen appearance and were unable to divide further, upon which they were left on an orbital shaker for six days at 28°C. The supernatants were then freed of cells by centrifugation (5000*g*, 10 min, 4°C) and used for protein production.

### 2.3. Recombinant protein expression and purification

**2.3.1. His-tagged protein expression and purification**. Sf9 insect cells were infected with His-tagged ACE2 baculovirus, at a 1:2000 dilution and left to agitate for 72 h. The culture volume was collected and supplemented with 40 m*M* HEPES and 300 m*M* NaCl, the pH was adjusted to 7.2 and 40 m*M* imidazole, 5 m*M* $MgCl_2$ and 0.5 m*M* $NiSO_4$ were added to the sample. The recombinant protein was purified from the supernatant using HisGraviTrap (Cytiva) columns. The eluted protein was loaded onto a HiPrep 26/10 Desalting column (Cytiva) to exchange the buffer to 20 m*M* Tris pH 8.0. Ion-exchange chromatography (IEC) was carried out on a HiTrap CaptoQ ImpRes column (Cytiva) to remove the remaining impurities. Purified ACE2 was concentrated in a 50 kDa 4 ml Amicon (Merck), aliquoted and frozen in liquid nitrogen for storage at −80°C.

**2.3.2. TwinStrep-tagged protein expression and purification**. TwinStrep-tagged ACE2 and RBD baculoviruses were used to infect Sf9 cells at a 1:2000 dilution for 72 h. Each culture medium was collected and recombinant protein was purified from the supernatant using a StrepTactin 4Flow 5 ml cartridge (Iba Lifesciences) and the buffers recommended by the manufacturer. Eluted samples were concentrated in 10 kDa 4 ml Amicons (Merck) and were further purified by size-exclusion chromatography (SEC) on a Superdex 200 10/300 GL column (Cytiva) in Tris-buffered saline (TBS) pH 7.4, 1 m*M* DTT buffer. For RBD proteins, 1 mM DTT was added to prevent dimerization. Protein samples were concentrated using 10 kDa Nanosep columns (Pall Corporation), aliquoted and frozen in liquid nitrogen for storage at −80°C.

### 2.4. Kinetic characterization using biolayer interferometry

The affinities of RBD wt, RBD Q498Y, RBD *β*, RBD K417N, RBD E484K, RBD 501Y, and the triple mutant RBD Q493K/Q498Y/P499T for ACE2 were tested by biolayer interferometry using a BLItz system (Sartorius). His-tagged ACE2 was immobilized onto Ni–NTA-coated biosensors (Sartorius) before association of increasing concentrations of each RBD for 120 s followed by dissociation using HEPES-buffered saline (HBS) pH 7.4 for 360 s. The obtained sensograms were corrected using a blank curve and fitted to a 1:1 Langmuir binding model using the *BLItz* software to characterize the binding affinities of each RBD to ACE2.

In another strategy to assess the affinities of the different RBDs for ACE2, RBD wt was immobilized onto activated amine-reactive sensors. The sensors were then dipped for 60 s into solutions containing 400 n*M* ACE2 or 400 n*M* ACE2 pre-incubated with equimolar amounts of RBD wt, RBD Q498Y and RBD *β* prior to dissociation with HBS pH 7.4 for a further 60 s. The sensograms obtained were corrected using a blank curve.

### 2.5. Binding-affinity and temperature-dependence characterization by ELISA

StrepTactin XT-coated microplates (Iba Lifesciences) were blocked for 2 h at room temperature using 10%(*w*/*v*) milk in phosphate-buffered saline (PBS), 0.2% Tween 20. The wells were washed with 100 m*M* Tris, 150 m*M* NaCl, 1 m*M* EDTA pH 8.0 [StrepTactin binding buffer (BB) 1×], 0.2% Tween 20 before immobilization of TwinStrep-tagged RBD wt, RBD Q498Y or RBD *β* at 1 µg $ml^{-1}$ for 30 min at room temperature. TwinStrep-tagged CIDRα1 at 1 µg $ml^{-1}$ was immobilized as a negative control to subtract nonspecific signal. The wells were then washed with BB 1×, 0.2% Tween 20, and 12×His-tagged ACE2 was added at concentrations ranging from 0 to 700 n*M*. 30 min incubations were performed at 4, 22 and 37°C to allow binding of the RBD proteins. After washing the wells with BB 1×, 0.2% Tween 20 (previously brought to 4, 22 or 37°C) to remove unbound ACE2, peroxidase-conjugated anti-polyhistidine antibody (Sigma–Aldrich, catalog No. A7058) at 1:10 000 dilution was added and incubated for a further 30 min at room temperature. After a final wash with BB 1×, 0.2% Tween 20, TMB One Substrate (Promega) was added to the wells and left for 10 min at room temperature in the dark before stopping the enzyme reaction with 2.0 *M* sulfuric acid. The absorbance at 450 nm was measured using an Epoch microplate spectrophotometer (Agilent) and the *Gen*5 software (version 2.09). Each data set was obtained in triplicate. Nonspecific signal measured from wells coated with a non-relevant protein (*Plasmodium falciparum* CIDRα1) was subtracted from each RBD value. The resulting absorbance values were then plotted, showing absorbance at 450 nm against (*y* axis) ACE2 concentration (*x* axis). Curves were fitted to a saturation, one-site specific binding model using *GraphPad Prism* 9.0 to calculate $K_d$ values for each ACE–RBD pair.

### 2.6. Neutralization ELISA

As in the ELISA experiments described above, StrepTactin XT-coated microplates were blocked for 2 h at room temperature using 10%(*w*/*v*) milk in PBS, 0.2% Tween 20. The wells were washed with StrepTactin BB 1×, 0.2% Tween 20

before immobilization of TwinStrep-tagged RBD wt (or CIDRα1 as a negative control) at 1 µg ml$^{-1}$ for 60 min at room temperature. The wells were then washed with Strep-Tactin BB 1×, 0.2% Tween 20 prior to addition of 10 µg ml$^{-1}$ 12×His-tagged ACE2 pre-incubated with increasing concentrations of either soluble TwinStrep tag-free RBD wt, RBD Q498Y or RBD *β* (in the range 0–3600 n*M* or 0–200 µg ml$^{-1}$). After incubation at room temperature for 60 min, the wells were washed again with StrepTactin BB 1×, 0.2% Tween 20, and peroxidase-conjugated anti-polyhistidine antibody was added at 1:10 000 dilution for a further 60 min. The wells were washed for a final time with StrepTactin BB 1×, 0.2% Tween 20 before incubation with TMB One Substrate for 10 min at room temperature in the dark. The enzymatic reaction was stopped using 2 *M* sulfuric acid and the absorbance at 450 nm was read using an Epoch microplate spectrophotometer (Agilent) and the *Gen*5 software (version 2.09). Each data set was obtained in triplicate.

Non-specific signal measured from CIDRα1-coated wells for each concentration of soluble RBD was subtracted from each specific value. The obtained absorbance values were normalized (with 0% absorbance being the lowest absorbance value and 100% absorbance being the highest value) and then plotted showing the percentage of absorbance at 450 nm on the *y* axis and the concentration of soluble RBD (n*M*) on the *x* axis. Curves were fitted to a dose–response inhibition, [inhibitor] versus normalized response model, equation using *GraphPad Prism* 9.0 to calculate $IC_{50}$ values for each RBD.

### 2.7. Crystallization of the RBD Q498Y–ACE2 complex

Sf9 insect cells were separately infected with TwinStrep-tagged RBD Q498Y and ACE2 (1:2000) viruses for 72 h. Recombinant proteins were purified from each collected supernatant by affinity chromatography using StrepTactin XT 4Flow 5 ml cartridges (Iba Lifesciences). Eluted RBD Q498Y and ACE2 were then concentrated and buffer-exchanged into TBS pH 7.4 using 10 and 50 kDa 4 ml Amicons (Merck), respectively. Tags were removed by overnight digestion with 3C protease (1:50) at 4°C. 3C-digested proteins were loaded onto StrepTactin XT 4Flow 1 ml gravity-flow columns (Iba Lifesciences) and collected in the flowthrough for quantification. Equimolar amounts of ACE2 and RBD Q498Y were then pooled together and the complex formed was purified using a Superdex 200 10/300 GL column (Cytiva) and TBS pH 7.4, 1 m*M* DTT buffer.

The purified RBD Q498Y–ACE2 complex was concentrated to 5.5 mg ml$^{-1}$ and screened against different crystallization conditions by the sitting-drop vapor-diffusion method. The best crystals were obtained in 0.1 *M* sodium phosphate pH 6.5, 12%(*w*/*v*) PEG 8000 and were captured, soaked in mother liquor containing 20% glycerol and cryo-cooled in liquid nitrogen.

### 2.8. X-ray diffraction data processing, structure determination and refinement

X-ray diffraction and data collection were performed on the BL13-XALOC beamline at the ALBA synchrotron facility. The diffraction data were reduced and integrated with *XDS* (Kabsch, 2010) and then merged and scaled with *AIMLESS* (Evans & Murshudov, 2013). The structure of the complex was solved via molecular replacement using *Phaser* (Read, 2001) with separate templates from the previously deposited coordinates of the RBD wt–ACE2 complex (Lan *et al.*, 2020). Restrained refinement of the structure was carried out with *REFMAC* (Kovalevskiy *et al.*, 2016) or *phenix.refine* (Adams *et al.*, 2010). The final molecule was generated after iterative cycles of manual building in *Coot* (Emsley *et al.*, 2010) and further refinement. The RBD Q498Y–ACE2 complex structure atomic coordinates and structure factors were deposited in the Protein Data Bank under the accession identifier 7p19.

### 2.9. Molecular-dynamics (MD) setup and protocol

Every PDB entry was pre-processed by the *tleap* program from the *Amber* package (*AmberTools*19 version) using the *ff19SBonlysc* force field (Tian *et al.*, 2020) for the protein and the OPC3BOX force field for the water solvent. The complex was soaked into a truncated solvated octahedral box with a minimum distance of 12 Å between any protein atom and the box edge, generating coordinates and parameter input files for MD simulation. The *prmtop* file was modified with hydrogen-mass repartitioning to allow an increased time step of 4 fs.

The equilibration protocol consisted of initial minimization and several steps of heating, and a gradual reduction of initial positional restraints. The production MD run was then performed at NVT, with periodic boundary conditions and Ewald summation for the long-range electrostatic interactions. Following molecular-dynamics simulations, the energy of binding was computed by two different methods: *MM-PBSA* and *pyDock* scoring function.

### 2.10. *MM-PBSA* scoring

The free energy of binding was estimated using *Molecular Mechanics Poisson–Boltzmann Surface Area* (*MM-PBSA*) as embedded in the *MMPBSA.py* module of *AMBER*18 (Ahmad *et al.*, 2021). The net energy of binding is estimated using the equation $\Delta G_{\rm mmpbsa} = \Delta G_{\rm ele} + \Delta G_{\rm vdw} + \Delta G_{\rm PB} + \Delta G_{\rm SA} - T\Delta S$.

The contribution of the polar solvation energy is calculated with the $\Delta G_{\rm PB}$ implicit solvent model, whereas the nonpolar part of the solvation energy is computed from the difference in solvent-accessible surface area (SASA) between the complex and the free components $\Delta G_{\rm SA}$. $\Delta G_{\rm ele}$ and $\Delta G_{\rm vdw}$ are the electrostatics and van der Waals components computed from the molecular-mechanics force field of *AMBER*, respectively, and $T\Delta S$ is the conformation entropy, which is usually neglected when the partners are similar.

*MM-PBSA* is usually computed for hundreds of unrelated conformations obtained during a molecular-dynamics simulation and the obtained complex binding energy values are averaged.

### 2.11. *pyDock* scoring

*pyDock* is a docking scoring approach (Cheng *et al.*, 2007), which uses an energy-based function to score the docking poses (*i.e.* different conformations) generated by a variety of sampling methods, such as *ZDOCK* or *FTDOCK*. Here, we applied the *pyDock bindEy* module to compute the binding energy of four different conformations of the complex between SARS-CoV-2 spike (WT and Q498Y mutant) and ACE2 sampled at different times during the molecular simulation.

The scoring function of *pyDock* is composed of electrostatics, desolvation energy and 10% van der Waals contributions: $\Delta G_{\rm pyDock} = \Delta G_{\rm ele} + \Delta G_{\rm desolv} + 0.1\Delta G_{\rm vdw}$.

## 3. Results

### 3.1. *In silico* prediction of RBD mutations that enhance binding to ACE2

Using a computational pipeline for high-throughput *in silico* mutagenesis (Rodrigues *et al.*, 2019), we explored substitutions in the RBD wild-type (RBD wt)–ACE2 interface that lead to a predicted increase in affinity by means of a reduction in the binding free-energy score. To predict the impact of such mutations, the method relies on the structure of a protein–protein template, the environment of the reference residue, the physicochemical properties of the replacement residue, the degree of evolutionary conservation and the thermodynamics of the protein–protein complex. An amino-acid substitution matrix focused on the wild-type RBD binding site was generated and used to plot a heatmap to evaluate variations in protein–protein binding energy, determined as the change in Gibbs free energy ($\Delta\Delta G$) upon single-residue mutagenesis. The mutational blueprint calculated for the RBD–ACE2 interface (Fig. 1*a*) showed a heterogeneous plot

**Figure 1**
Mutational blueprint of the RBD–ACE2 interface. (*a*) Heatmap plotting changes in the predicted binding affinity of RBD wt to ACE2. The RBD binding interface was screened *in silico* (Rodrigues *et al.*, 2019) for amino-acid substitutions leading to a predicted enhanced affinity for ACE2. The working template was obtained from the X-ray coordinates deposited in the Protein Data Bank under accession code 6m0j. Those mutations that increase the affinity for ACE2 have a positive value and are highlighted in blue, while those that decrease the affinity of RBD for ACE2 have a negative value and are shown in red. (*b*) Overall cartoon representation of the RBD wt–ACE2 crystal structure (left panel) with position 498 in RBD wt and the surrounding residues highlighted in yellow. The right panel shows a closer image of Gln498 in RBD wt. The dashed line represents a likely (3.4 Å) hydrogen bond between Gln498 of RBD wt and Gln42 of ACE2.

whereby position 475 in RBD, corresponding to an alanine, leads to the most favorable substitutions in terms of affinity. In contrast, Tyr489 is apparently the least favorable residue to enhance binding to ACE2 upon substitution. Looking at the chemical nature of the replacement amino acid, substitutions to Tyr and Pro result in the most and least beneficial variations, respectively. Here, we focused on the substitution with the greatest score, Q498Y, which yields a predicted affinity change ($\Delta\Delta G$) of 2.0 kcal mol$^{-1}$. Gln498 is located within the RBD–ACE2 binding interface, in the RBD region near Tyr41 and Gln42 in the ACE2 N-terminal $\alpha$-helix, with which it establishes polar and van der Waals contacts (Fig. 1*b*). More specifically, there is a putative hydrogen bond to the side chain of Gln42 at a distance of 3.4 Å. van der Waals contacts are also present and mediate interactions with Tyr41 and Gln42 in ACE2. Thus, the crystal structure supports the relevance of this residue in the overall binding of the RBD to ACE2.

### 3.2. A single Q→Y substitution at position 498 of RBD confers improved binding to ACE2

In order to investigate the impact of the Q498Y mutation on the binding kinetics with ACE2, we produced recombinant human ACE2, RBD wt and RBD Q498Y in Sf9 cells. As an additional reference, we also expressed the RBD of the $\beta$ variant of concern for purposes of comparison. The purified proteins were used to carry out biolayer interferometry assays to accurately characterize their binding affinities. We first captured ACE2 on the surface of an $Ni^{2+}$ sensor through a 12×His C-terminal tag. Increasing concentrations of RBD wt, RBD Q498Y or RBD $\beta$ were then loaded and the association and dissociation curves were monitored in real time (Fig. 2 and Supplementary Fig. S2). We monitored remarkably stable binding for all three RBD forms tested, where a tendency to a modestly slower dissociation rate could be noticed for the Q498Y and $\beta$ forms of the RBD (Fig. 2 and Table 1). A more accurate comparison was obtained by determining the kinetic constants of each interaction. The data could be fitted to a 1:1 Langmuir model with coefficients of determination $R^2$ above 0.99 in all cases. All three RBD forms bound ACE2 with very high affinity, yielding $K_d$ values in the near-subnanomolar range (Table 1). Among the three RBD forms tested, RBD Q498Y showed the fastest association rate, but with a $k_{on}$ value very similar to those of RBD wt and RBD $\beta$ (Table 1).

**Table 1**
Kinetic constants determined by biolayer interferometry.

The association and dissociation constants, $k_{on}$ and $k_{off}$, respectively, are shown as well as the overall $K_d$ ($k_{off}/k_{on}$) value. The goodness of fit of the binding data to a 1:1 Langmuir model is represented by the $R^2$ or coefficient of determination. Each experiment was run in triplicate. N.D., not determined due to negligible dissociation.

| RBD | $k_{on} \times 10^5$ ($M^{-1}$ s$^{-1}$) | $k_{off} \times 10^{-4}$ (s$^{-1}$) | $K_d$ (n$M$) | $R^2$ |
|---|---|---|---|---|
| wt | 1.3 ± 0.3 | 11.9 ± 5.2 | 8.9 ± 2.1 | 0.9980 |
| Q498Y | 1.6 ± 0.3 | 4.5 ± 2.5 | 3.0 ± 2.2 | 0.9975 |
| $\beta$ | 1.2 ± 0.1 | 2.9 ± 0.4 | 2.4 ± 0.5 | 0.9976 |
| K417N | 1.0 ± 0.2 | 13.7 ± 1.7 | 14.6 ± 3.8 | 0.9968 |
| E484K | 1.3 ± 0.4 | 9.3 ± 5.7 | 6.8 ± 2.4 | 0.9967 |
| N501Y | 1.2 ± 0.1 | N.D. | N.D. | 0.9973 |
| Q493K+Q498Y+P499T | 1.3 ± 0.1 | 3.4 ± 0.7 | 2.7 ± 0.8 | 0.9972 |

**Figure 2**
RBD Q498Y–ACE2 binding kinetics determined by biolayer interferometry (BLI). (*a*) ACE2 was captured on the surface of an Ni–NTA sensor and pulsed for 2 min with increasing concentrations of RBD wt, RBD Q498Y or RBD $\beta$ in running buffer (20 m$M$ HEPES pH 7.4, 150 m$M$ NaCl). The association and dissociation data were monitored and fitted to a 1:1 Langmuir model for the calculation of binding kinetics constants ($k_{on}$, $k_{off}$ and $K_d$) and assessment of the goodness of fit ($R^2$). The signal corresponding to the buffer was subtracted prior to any calculations. Colored lines represent experimental raw data and black lines the binding kinetics fittings.

The main difference between RBD Q498Y and RBD wt was in the dissociation rate, where RBD Q498Y showed a ~2.5-fold lower $k_{off}$ compared with that of RBD wt. Thus, RBD Q498Y resulted in an overall ~2.5-fold tighter binding. The RBD $\beta$ variant represented the most stable binder, with a $k_{off}$ value of $2.8 \times 10^{-4}$ $s^{-1}$, *i.e.* a dissociation rate ~2 and ~4.5-fold slower than those of RBD Q498Y and RBD wt, respectively. Nevertheless, and despite the three amino-acid substitutions, the overall binding affinity of the RBD $\beta$ form is only 1.6 times stronger than that of RBD Q498Y (Fig. 2, Table 1). This results in a relative change in affinity of 1.4-fold per amino acid replaced, while the single Q498Y replacement confers a 2.5-fold increase in binding affinity.

To further confirm this trend, we undertook an additional but indirect BLI approach whereby RBD wt was covalently immobilized on the surface of a sensor. ACE2 at a concentration of 400 n*M* was then pulsed and the binding curves were compared with similar concentrations of ACE2 but previously incubated with stoichiometric amounts of either RBD wt, RBD Q498Y or RBD $\beta$ (Fig. 3*a*). In other words, we compared the binding affinities using an alternative approach in solution. This alternative strategy allowed us to confirm the stronger binding between ACE2 and RBD Q498Y when compared with RBD wt, as we noticed a stronger competition in the association of ACE2 to surface RBD wt when the receptor was pre-incubated with the mutant RBD Q498Y. As in the direct assay, the RBD $\beta$ variant showed the strongest binding, as mirrored in the strongest inhibition of ACE2 binding to surface-immobilized RBD wt (Fig. 3*b*).

We performed additional binding studies in a plate ELISA-like binding assay with surface-immobilized RBD wt proteins and ACE2 in solution. RBD wt competitors were added in solution and the $IC_{50}$ values were determined. RBD Q498Y yielded the strongest competition ($IC_{50}$ = 119 n*M*), followed by RBD $\beta$ ($IC_{50}$ = 121 n*M*) and RBD wt ($IC_{50}$ = 168 n*M*). Together, these results support the RBD Q498Y substitution conferring stronger binding to ACE2.

### 3.3. Structural bases of the enhanced affinity conferred by the Q498Y substitution

To explore the structural bases for this enhanced affinity, we prepared a complex between RBD Q498Y and ACE2. Incubation of RBD Q498Y with ACE2 showed a sharp shift in the retention time of the protein via size-exclusion chromatography (Fig. 4*a*). We then screened >750 crystallization conditions to obtain crystals of RBD Q498Y complexed with ACE2 (Fig. 4*a*, inset). Crystals of the RBD Q498Y–ACE2 complex appeared with 0.1 *M* sodium phosphate pH 6.5, 12% PEG 8000. A full data set was collected to 3.25 Å resolution and was processed in space group $P2_12_12_1$ (Table 2). Initial phases were calculated with the molecular-replacement method and yielded two RBD Q498Y–ACE2 complexes per asymmetric unit. Unlike the wild-type RBD–ACE2 complex (PDB entry 6m0j), which crystallized with one complex per asymmetric unit, the RBD Q498Y–ACE2 crystallization process led to two complexes per asymmetric unit (Fig. 4*b*). The overall docking mode is almost identical to that of the complex with RBD wt (Fig. 5*a*), with a root-mean-square deviation of 0.916 upon alignment on ACE2.

The binding footprint is preserved with that of RBD wt and shows an elongated interface where RBD Q498Y interacts primarily with the N-terminal $\alpha$-helix of ACE2 (Fig. 5, Table 3). The buried surface area (BSA) of the interaction covers 820 and 837 Å$^2$ in each complex structure in the asymmetric unit. As for the complex with RBD wt, a strong electron-density signal is also found for a putative $Zn^{2+}$ ion coordinated by His374, His378, Glu375 and Glu402 in ACE2. The electron

**Figure 3**
Indirect assessment of the Q498Y substitution in the binding affinity to ACE2. (*a*) ACE2 at a concentration of 400 n*M*, either free (blue line) or pre-incubated in solution with 400 n*M* RBD wt, RBD Q498Y or RBD $\beta$, was loaded over the RBD wt-coated surface and the binding profile was monitored. (*b*) Inhibition of the coated SARS-CoV-2 RBD wt–ACE2 interaction by soluble RBD wt, RBD Q498Y or RBD $\beta$ measured by neutralization in an ELISA-like plate assay. The graph shows the reduction of ACE2 binding to surface-immobilized RBD as the concentration of competitor in solution increases. Binding is measured as the absorbance at 450 nm (*y* axis) and is plotted against the concentration of each competitor (*x* axis). Each point represents mean absorbance percentage values ± standard deviation (SD). Normalized absorbance values (with 0% being the lowest value and 100% being the highest) were fitted to a concentration of inhibitor versus normalized response equation using *GraphPad Prism* 9.0 to obtain the curves and calculate the $IC_{50}$ values for each tested RBD.

**Table 2**
X-ray data-collection and refinement statistics.

Valeus in parentheses are for the highest resolution shell.

| | |
|---|---|
| Resolution range (Å) | 93.99–3.24 (3.35–3.24) |
| Space group | $P2_12_12_1$ |
| $a$, $b$, $c$ (Å) | 60.591, 165.037, 228.678 |
| $\alpha$, $\beta$, $\gamma$ (°) | 90, 90, 90 |
| Total reflections | 202345 (20961) |
| Unique reflections | 37446 (3704) |
| Multiplicity | 5.4 (5.7) |
| Completeness (%) | 99.43 (99.78) |
| Mean $I/\sigma(I)$ | 7.87 (1.19) |
| Wilson $B$ factor (Å$^2$) | 85.19 |
| $R_{\rm merge}$ | 0.2136 (1.508) |
| $R_{\rm meas}$ | 0.2365 (1.661) |
| $R_{\rm p.i.m.}$ | 0.09988 (0.6879) |
| $CC_{1/2}$ | 0.995 (0.651) |
| $R_{\rm work}$ | 0.245 (0.384) |
| $R_{\rm free}$ | 0.292 (0.408) |
| R.m.s.d., bond angles | 0.002 |
| R.m.s.d., angles | 0.53 |
| Ramachandran favored (%) | 95.23 |
| Ramachandran outliers (%) | 0.07 |

density surrounding position 498 in the RBD allowed us to accurately locate Tyr498 in the RBD (Fig. 5*b*). More specifically, the Tyr498 side chain projects towards ACE2 in a similar manner to that of Gln498 in the complex structure with the RBD wt counterpart (Figs. 6*a*–6*c*). Despite the similar orientation, this structural arrangement and the bulkier side chain of tyrosine in RBD Q498Y imply important differences with respect to Gln498 in RBD wt. First, in the RBD wt structure Gln498 contacts Gln42 of ACE2 through a single 3.4 Å hydrogen bond (Fig. 6*a*). In contrast, Tyr498 establishes, on one side, a shorter (2.7 Å) hydrogen bond to the side chain of Gln42. Further, Tyr498 locks Tyr449, also in the RBD, through a 2.4 Å side chain–side chain hydrogen bond. This interaction slightly alters the hydrogen-bonding distances between RBD Tyr449 and the side-chain O atoms of Asp38 in ACE2; we observe distances of 3.0 and 2.5 Å versus 2.7 and 3.2 Å in the complex with RBD wt.

Importantly, nonpolar interactions, such as van der Waals contacts, are present at a greater frequency in the case of the RBD Q498Y complex (Fig. 6*b*). The relevance of these contacts is mirrored by Tyr498 engaging up to four different amino acids in ACE2: Asp38, Tyr41, Gln42 and Lys353. Of these, Asp38, Tyr41 and Gln42 are located on the main interacting helix of ACE2 in the N-terminal region, while Lys353 belongs to a different domain found in the RBD–ACE2 interface. On the contrary, Gln498 lacks nonpolar contacts with Asp38 and Lys353. Moreover, in addition to these interactions, the presence of Tyr498 enables $\pi$–$\pi$ stacking interactions with the side chain of Tyr42 in ACE2 (Fig. 6*c*).

As mentioned earlier, we found that the $\beta$ variant showed the highest affinity for ACE2. This variant harbors K417N, E484K and N501Y substitutions. From a structural point of view, Lys417 establishes strong salt bridges with Asp30 in ACE2 that cannot be maintained by Asn417 in the RBD $\beta$ variant (Supplementary Fig. S3). Regarding position 484, no contacts are found for either the Glu or Lys residues in the wild-type or $\beta$ variant RBDs, respectively. Asn501 in wild-type RBD makes few contacts with Tyr41, while its counterpart Tyr501 in RBD $\beta$ provides an abundant number of van der Waals contacts (with Tyr41 and Lys353 of ACE2) and, additionally, $\pi$–$\pi$ interactions (with Tyr41 of ACE2). We also tested each of these mutations individually via biolayer interferometry (Fig. 7 and Table 1). In our binding analysis with ACE2 immobilized via a 12×His tag, we determined $K_{\rm d}$ affinity values of 16.8 and 8.1 n*M* for the RBD K417N and E484K mutants, respectively, while the N501Y mutant yielded undetectable dissociation and therefore produced the greatest impact.

Altogether, Tyr498 contributes to ACE2 binding by promoting a more populated and extensive set of interactions via which the RBD grips ACE2 by engaging different domains.

### 3.4. Computation of the complex binding energy

In order to analyze the energetic impact of the SARS-CoV-2 spike mutation on its interaction with ACE2, we performed 500 ns molecular-dynamics simulations of the complex between SARS-CoV-2 spike RBD (wt and Q498Y) and ACE2. Based on 1000 snapshots extracted from the last 20 ns

**Figure 4**
Formation of the RBD Q498Y–ACE2 complex. (*a*) The elution profile of RBD Q498Y alone (orange trace) or complexed with ACE2 (blue trace) was monitored to track the proper formation of the complex through size-exclusion chromatography. The inset shows an image of crystals of the RBD Q498Y–ACE2 complex. mAU, absorbance units (×10$^{-3}$). (*b*) Composition of the asymmetric unit in the RBD Q498Y–ACE2 crystal. All RBD and ACE2 molecules are shown as individual colored cartoons accompanied by a $2F_{\rm o} - F_{\rm c}$ electron-density map (gray) contoured at the 1.0$\sigma$ level.

**Table 3**
Interatomic contacts between RBD Q498Y and ACE2.

van der Waals interactions, salt bridges and hydrogen bonds are listed based on distance cutoffs of 4, 4.5 and 3.4 Å, respectively.

| RBD | ACE2 | Distance (Å) | Bond type |
|---|---|---|---|
| Lys417 C$^{\varepsilon}$ | Asp30 C$^{\gamma}$ | 4.0 | van der Waals |
| Lys417 C$^{\varepsilon}$ | Asp30 O$^{\delta1}$ | 3.9 | van der Waals |
| Lys417 C$^{\varepsilon}$ | Asp30 O$^{\delta2}$ | 3.2 | van der Waals |
| Lys417 N$^{\zeta}$ | Asp30 C$^{\gamma}$ | 3.9 | van der Waals |
| Lys417 N$^{\zeta}$ | Asp30 O$^{\delta2}$ | 2.8 | Salt bridge |
| Lys417 N$^{\zeta}$ | Asp30 O$^{\delta1}$ | 4.3 | Salt bridge |
| Tyr449 C$^{\varepsilon1}$ | Asp38 C$^{\gamma}$ | 3.6 | van der Waals |
| Tyr449 C$^{\varepsilon1}$ | Asp38 O$^{\delta2}$ | 3.2 | van der Waals |
| Tyr449 C$^{\varepsilon1}$ | Asp38 O$^{\delta1}$ | 3.4 | van der Waals |
| Tyr449 C$^{\zeta}$ | Asp38 C$^{\gamma}$ | 3.8 | van der Waals |
| Tyr449 C$^{\zeta}$ | Asp38 O$^{\delta2}$ | 3.4 | van der Waals |
| Tyr449 C$^{\zeta}$ | Asp38 O$^{\delta1}$ | 3.4 | van der Waals |
| Tyr449 O$^{\eta}$ | Asp38 C$^{\gamma}$ | 3.1 | van der Waals |
| Tyr449 O$^{\eta}$ | Asp38 O$^{\delta2}$ | 3.0 | Hydrogen bond |
| Tyr449 O$^{\eta}$ | Asp38 O$^{\delta1}$ | 2.5 | Hydrogen bond |
| Tyr449 O$^{\eta}$ | Gln42 C$^{\gamma}$ | 4.0 | van der Waals |
| Tyr449 O$^{\eta}$ | Gln42 C$^{\delta}$ | 3.5 | van der Waals |
| Tyr449 O$^{\eta}$ | Gln42 O$^{\varepsilon1}$ | 3.8 | van der Waals |
| Tyr449 O$^{\eta}$ | Gln42 N$^{\varepsilon2}$ | 3.4 | Hydrogen bond |
| Leu455 C$^{\delta1}$ | Asp30 O$^{\delta2}$ | 3.9 | van der Waals |
| Phe456 C$^{\delta1}$ | Thr27 C$^{\gamma2}$ | 3.8 | van der Waals |
| Phe456 C$^{\varepsilon1}$ | Thr27 C$^{\gamma2}$ | 3.5 | van der Waals |
| Phe456 C$^{\varepsilon1}$ | Asp30 O$^{\delta2}$ | 3.3 | van der Waals |
| Phe456 C$^{\zeta}$ | Thr27 C$^{\gamma2}$ | 4.0 | van der Waals |
| Ala475 O | Gln24 C$^{\gamma}$ | 3.4 | van der Waals |
| Phe486 C$^{\delta1}$ | Tyr83 O$^{\eta}$ | 3.9 | van der Waals |
| Phe486 C$^{\varepsilon1}$ | Met82 C$^{\beta}$ | 3.6 | van der Waals |
| Phe486 C$^{\varepsilon1}$ | Tyr83 C$^{\varepsilon1}$ | 3.9 | van der Waals |
| Phe486 C$^{\varepsilon1}$ | Tyr83 C$^{\zeta}$ | 3.6 | van der Waals |
| Phe486 C$^{\varepsilon1}$ | Tyr83 O$^{\eta}$ | 3.5 | van der Waals |
| Phe486 C$^{\zeta}$ | Tyr83 C$^{\varepsilon1}$ | 3.9 | van der Waals |
| Asn487 C$^{\gamma}$ | Gln24 O$^{\varepsilon1}$ | 3.3 | van der Waals |
| Asn487 C$^{\gamma}$ | Tyr83 O$^{\eta}$ | 3.4 | van der Waals |
| Asn487 O$^{\delta1}$ | Gln24 O$^{\varepsilon1}$ | 3.6 | van der Waals |
| Asn487 O$^{\delta1}$ | Gln24 C$^{\beta}$ | 3.9 | van der Waals |
| Asn487 O$^{\delta1}$ | Gln24 C$^{\gamma}$ | 3.8 | van der Waals |
| Asn487 O$^{\delta1}$ | Tyr83 C$^{\varepsilon1}$ | 3.7 | van der Waals |
| Asn487 O$^{\delta1}$ | Tyr83 C$^{\zeta}$ | 3.7 | van der Waals |
| Asn487 O$^{\delta1}$ | Tyr83 O$^{\eta}$ | 2.8 | Hydrogen bond |
| Asn487 N$^{\delta2}$ | Gln24 C$^{\delta}$ | 3.5 | van der Waals |
| Asn487 N$^{\delta2}$ | Gln24 O$^{\varepsilon1}$ | 2.4 | Hydrogen bond |
| Asn487 N$^{\delta2}$ | Tyr83 C$^{\varepsilon1}$ | 4.0 | van der Waals |
| Asn487 N$^{\delta2}$ | Tyr83 O$^{\eta}$ | 3.9 | van der Waals |
| Tyr489 C$^{\delta2}$ | Lys31 C$^{\gamma}$ | 4.0 | van der Waals |
| Tyr489 C$^{\varepsilon1}$ | Thr27 C$^{\gamma2}$ | 3.9 | van der Waals |
| Tyr489 O$^{\eta}$ | Phe28 C$^{\beta}$ | 3.9 | van der Waals |
| Tyr489 O$^{\eta}$ | Tyr83 O$^{\eta}$ | 3.3 | Hydrogen bond |
| Tyr489 O$^{\eta}$ | Phe28 N | 3.8 | van der Waals |
| Tyr489 O$^{\eta}$ | Phe28 C$^{\alpha}$ | 3.7 | van der Waals |
| Gln493 N$^{\varepsilon2}$ | His34 C$^{\beta}$ | 3.5 | van der Waals |
| Gln493 N$^{\varepsilon2}$ | His34 C | 3.7 | van der Waals |
| Gln493 N$^{\varepsilon2}$ | Glu35 N | 3.9 | van der Waals |
| Gln493 N$^{\varepsilon2}$ | His34 O | 3.7 | van der Waals |
| Gly496 C$^{\alpha}$ | Lys353 N$^{\zeta}$ | 3.7 | van der Waals |
| Gly496 C | Lys353 N$^{\zeta}$ | 3.8 | van der Waals |
| Gly496 O | Lys353 C$^{\delta}$ | 3.9 | van der Waals |
| Gly496 O | Lys353 C$^{\varepsilon}$ | 3.8 | van der Waals |
| Gly496 O | Lys353 N$^{\zeta}$ | 3.1 | Hydrogen bond |
| Tyr498 C$^{\beta}$ | Tyr41 C$^{\varepsilon2}$ | 3.9 | van der Waals |
| Tyr498 C$^{\gamma}$ | Tyr41 C$^{\varepsilon2}$ | 3.6 | van der Waals |
| Tyr498 C$^{\delta1}$ | Tyr41 C$^{\delta2}$ | 3.8 | van der Waals |
| Tyr498 C$^{\delta1}$ | Tyr41 C$^{\varepsilon2}$ | 3.4 | van der Waals |
| Tyr498 C$^{\varepsilon1}$ | Lys353 N$^{\zeta}$ | 3.8 | van der Waals |
| Tyr498 C$^{\varepsilon1}$ | Tyr41 C$^{\delta2}$ | 3.8 | van der Waals |
| Tyr498 C$^{\varepsilon1}$ | Tyr41 C$^{\varepsilon2}$ | 3.9 | van der Waals |
| Tyr498 C$^{\varepsilon2}$ | Gln42 O$^{\varepsilon1}$ | 4.0 | van der Waals |
| Tyr498 C$^{\zeta}$ | Gln42 O$^{\varepsilon1}$ | 3.6 | van der Waals |
| Tyr498 O$^{\eta}$ | Asp38 O$^{\delta1}$ | 3.8 | van der Waals |
| Tyr498 O$^{\eta}$ | Gln42 C$^{\gamma}$ | 3.8 | van der Waals |
| Tyr498 O$^{\eta}$ | Gln42 C$^{\delta}$ | 3.1 | van der Waals |
| Tyr498 O$^{\eta}$ | Gln42 O$^{\varepsilon1}$ | 2.7 | Hydrogen bond |
| Tyr498 O$^{\eta}$ | Gln42 N$^{\varepsilon2}$ | 3.7 | van der Waals |
| Thr500 C | Tyr41 O$^{\eta}$ | 3.6 | van der Waals |
| Thr500 C | Asp355 O$^{\delta2}$ | 3.8 | van der Waals |
| Thr500 O | Tyr41 O$^{\eta}$ | 3.7 | van der Waals |
| Thr500 O | Asp355 C$^{\gamma}$ | 3.4 | van der Waals |
| Thr500 O | Asp355 O$^{\delta1}$ | 3.8 | van der Waals |
| Thr500 O | Asp355 O$^{\delta2}$ | 3.2 | Hydrogen bond |
| Thr500 O | Asn330 N$^{\delta2}$ | 4.0 | van der Waals |
| Thr500 C$^{\beta}$ | Tyr41 O$^{\eta}$ | 3.6 | van der Waals |
| Thr500 C$^{\beta}$ | Arg357 N$^{\eta2}$ | 3.7 | van der Waals |
| Thr500 C$^{\beta}$ | Asp355 O$^{\delta2}$ | 3.5 | van der Waals |
| Thr500 C$^{\beta}$ | Asn330 N$^{\delta2}$ | 3.9 | van der Waals |
| Thr500 O$^{\gamma1}$ | Tyr41 C$^{\zeta}$ | 3.7 | van der Waals |
| Thr500 O$^{\gamma1}$ | Tyr41 O$^{\eta}$ | 2.5 | Hydrogen bond |
| Thr500 O$^{\gamma1}$ | Arg357 N$^{\eta2}$ | 3.7 | van der Waals |
| Thr500 O$^{\gamma1}$ | Asp355 O$^{\delta2}$ | 3.4 | Hydrogen bond |
| Thr500 C$^{\gamma2}$ | Arg357 N$^{\eta2}$ | 3.9 | van der Waals |
| Asn501 N | Tyr41 O$^{\eta}$ | 3.6 | van der Waals |
| Asn501 C$^{\alpha}$ | Lys353 O | 3.8 | van der Waals |
| Asn501 C$^{\alpha}$ | Tyr41 O$^{\eta}$ | 3.9 | van der Waals |
| Asn501 C | Lys353 O | 3.8 | van der Waals |
| Ans501 O$^{\delta1}$ | Lys353 C$^{\delta}$ | 3.8 | van der Waals |
| Ans501 O$^{\delta1}$ | Lys353 C$^{\beta}$ | 3.7 | van der Waals |
| Ans501 O$^{\delta1}$ | Tyr41 C$^{\varepsilon2}$ | 3.2 | van der Waals |
| Ans501 O$^{\delta1}$ | Tyr41 C$^{\zeta}$ | 3.2 | van der Waals |
| Asn501 O$^{\delta1}$ | Tyr41 O$^{\eta}$ | 3.2 | Hydrogen bond |
| Asn501 N$^{\delta2}$ | Lys353 C$^{\delta}$ | 3.9 | van der Waals |
| Gly502 N | Lys353 C | 3.9 | van der Waals |
| Gly502 N | Lys353 O | 2.8 | Hydrogen bond |
| Gly502 N | Gly354 C | 3.8 | van der Waals |
| Gly502 N | Gly354 O | 3.6 | van der Waals |
| Gly502 C$^{\alpha}$ | Lys353 O | 3.6 | van der Waals |
| Gly502 C$^{\alpha}$ | Gly354 C$^{\alpha}$ | 3.9 | van der Waals |
| Gly502 C$^{\alpha}$ | Gly354 C | 3.8 | van der Waals |
| Gly502 C$^{\alpha}$ | Gly354 O | 3.3 | van der Waals |
| Tyr505 C$^{\beta}$ | Lys353 O | 3.6 | van der Waals |
| Tyr505 C$^{\gamma}$ | Lys353 C$^{\alpha}$ | 3.7 | van der Waals |
| Tyr505 C$^{\gamma}$ | Lys353 C | 3.8 | van der Waals |
| Tyr505 C$^{\gamma}$ | Lys353 O | 3.8 | van der Waals |
| Tyr505 C$^{\gamma}$ | Lys353 C$^{\beta}$ | 4.0 | van der Waals |
| Tyr505 C$^{\delta2}$ | Lys353 C$^{\alpha}$ | 3.5 | van der Waals |
| Tyr505 C$^{\delta2}$ | Lys353 C | 3.3 | van der Waals |
| Tyr505 C$^{\delta2}$ | Lys353 O | 3.7 | van der Waals |
| Tyr505 C$^{\delta2}$ | Gly354 N | 3.6 | van der Waals |
| Tyr505 C$^{\varepsilon2}$ | Lys353 C$^{\alpha}$ | 3.7 | van der Waals |
| Tyr505 C$^{\varepsilon2}$ | Lys353 C | 3.9 | van der Waals |
| Tyr505 C$^{\varepsilon2}$ | Gly354 N | 3.9 | van der Waals |
| Tyr505 C$^{\zeta}$ | Glu37 O$^{\varepsilon2}$ | 3.9 | van der Waals |
| Tyr505 O$^{\eta}$ | Glu37 C$^{\delta}$ | 3.8 | van der Waals |
| Tyr505 O$^{\eta}$ | Glu37 O$^{\varepsilon1}$ | 3.9 | van der Waals |
| Tyr505 O$^{\eta}$ | Glu37 O$^{\varepsilon2}$ | 3.6 | van der Waals |

of molecular dynamics, and following the suggested strategy from a recent study on SARS-CoV-2 spike protein–ACE2 binding affinities (Piplani *et al.*, 2021), we estimated the interaction free energy using the *MM-PBSA* method (see Table 4). The results indicated that the RBD Q498Y mutation favored the interaction with ACE2, which is in agreement with our experimental results.

To further confirm these results, we additionally computed the binding energy of the complexes of ACE2 with RBD wt and RBD Q498Y using the *pyDock* scoring function (Cheng *et*

*al.*, 2007), which is composed of van der Waals, electrostatics and desolvation energy terms, with atomic parameters adjusted for rigid-body protein–protein docking. This scoring function can be used to estimate the impact of mutations on the binding energy of a protein–protein complex. Here, we computed the *pyDock* energy for four different conformations extracted from the molecular-dynamics simulation. The *pyDock* results (Table 5) clearly indicate that the Q498Y RBD mutant provides a higher affinity interaction than RBD wt, which is in agreement with our experimental results. While *pyDock* does not calculate an absolute binding free energy, since it does not include configurational entropy, we believe that the substantial difference between the binding energy of the WT and the mutant is indeed indicative of their relative interface stability, once again supporting our experimental findings.

### 3.5. Impact of temperature on RBD–ACE2 interactions

The effect of temperature was examined in an ELISA-like plate-binding assay for the three RBD proteins. For this, we first captured either RBD wt, RBD Q498Y or RBD $\beta$ on StrepTactin-coated microplates through the TwinStrep tag. Increasing amounts of 12×His-tagged ACE2 were then incubated and the binding was assessed by the absorbance generated with an anti-His-tag antibody conjugated with peroxidase (further details are available in Section 2). Here, we found that RBD Q498Y showed the strongest binding at all temperatures tested (4, 22 and 37°C),

**Figure 5**
Structural features of the RBD Q498Y–ACE2 complex. (*a*) Comparison of the overall complex structures of ACE2 complexed with RBD wt (image prepared with the atomic coordinates deposited in the Protein Data Bank; accession code 6m0j) and RBD Q498Y, as indicated in the figure. To compare the overall docking similarity between the two complexes, the RBD Q498Y–ACE2 structure was superposed on the $\alpha$ carbons of ACE2 in complex with RBD wt (PDB entry 6m0j). The structural alignment and all figures were generated in *PyMOL* (version 2.5.2). The middle panel in (*a*) shows both complexes structurally aligned on ACE2. The RBD and ACE2 proteins are shown as cartoons in pale yellow and gray, respectively. For purposes of comparison, the RBD Q498Y–ACE2 complex is shown in blue in the middle panel. To ease visualization, position 498 is highlighted with dots and the zinc(II) ions are displayed as purple spheres. (*b*) $2F_o - F_c$ electron-density map (contour level 1.0$\sigma$) surrounding position 498 of RBD in the refined structure of the RBD Q498Y–ACE2 complex shown as a blue mesh.

followed by RBD $\beta$ and RBD wt (Fig. 8 and Table 6). This trend was accentuated at 37°C. We also observed that the binding was reduced as the temperature increased, with RBD $\beta$ being the variant with the most evident temperature dependence. Thus, these studies show that the RBD–ACE2 interaction is more favorable at lower temperatures.

### 3.6. Q498Y concomitant with other substitutions

In order to assess Q498Y concomitant with other substitutions, we produced the triple mutant Q493K/Q498Y/P499T, as Leist and coworkers observed that these simultaneous replacements in the SARS-CoV-2 spike protein are associated with lethality in a murine model of infection (Leist *et al.*, 2020).

**Figure 6**
Comparison of the interatomic contacts between RBD and ACE2 in the wild-type and Q498Y structures. (*a*) Interatomic polar interactions are shown as blue dashed lines. The cutoffs for van der Waals interactions and hydrogen bonds are set to 4 and 3.4 Å, respectively. Interacting residues are depicted as sticks. (*b*) Interatomic nonpolar interactions are indicated as dashed lines and the interacting residues are highlighted as sticks. (*c*) $\pi$–$\pi$ stacking interaction established between the aromatic rings of Tyr498 in RBD and Tyr41 in ACE2.

**Table 4**
Interaction free-energy values calculated by *MM-PBSA* based on conformations extracted from MD of wt and mutated complexes.

| | Average *MM-PBSA*† | SEM‡ |
|---|---|---|
| WT | −70.36 | 0.23 |
| Q498Y | −73.28 | 0.23 |

† Averaged interaction free-energy values computed by *MM-PBSA* from 1000 snapshots extracted from the last 20 ns of a 500 ns trajectory. All values are expressed in kcal mol$^{-1}$ units. ‡ Standard error (SEM) computed for the values obtained from the 1000 snapshots.

**Table 5**
Binding-energy values computed with *pyDock* based on conformations extracted from MD of wt and mutated complexes.

| MD time | 3 ns† | 20 ns† | 200 ns† | 400 ns† | AVG‡ | SEM‡ |
|---|---|---|---|---|---|---|
| WT | −32.08 | −30.18 | −36.11 | −27.22 | −31.39 | 1.86 |
| Q498Y | −47.53 | −48.52 | −42.59 | −47.19 | −46.45 | 1.32 |

† The *pyDock* binding energy was computed for four WT and mutated complex conformations sampled during the simulation. All values are expressed in kcal mol$^{-1}$

**Figure 7**
Kinetics and binding-affinity analysis of ACE2 with individual mutations found in the SARS-CoV-2 $\beta$ VOC spike RBD. Biolayer interferometry analysis using ACE2 captured on the surface of an Ni–NTA sensor, as described in Fig. 2. Increasing concentrations of RBD mutants K417N, E484K and N501Y were exposed to ACE2 in 20 m*M* HEPES pH 7.4, 150 m*M* NaCl. Red, experimental data; black, fitting of the experimental data to a 1:1 Langmuir model.

**Table 6**
Effect of temperature on the binding kinetics of RBD Q498Y to human ACE2.

The affinity $K_d$ values for the binding of ACE2 to plate-immobilized RBD Q498Y are shown. For purposes of comparison, the binding was also tested with RBD wt and RBD $\beta$.

| $K_d$ (n*M*) | 4°C | RT | 37°C |
|---|---|---|---|
| WT | 17 | 29 | 58 |
| Q498Y | 9 | 12 | 16 |
| $\beta$ | 14 | 25 | 73 |

Our binding studies showed that this triple substitution confers a modestly higher binding due to a slower dissociation rate, as observed from the $k_{off}$ values (Supplementary Fig. S3 and Table 1). Therefore, our results indicate that Q493K and P499T mutations concomitant with Q498Y do not have a critical impact on binding human ACE2.

## 4. Discussion

In the last two decades humankind has been exposed to the emergence of numerous coronaviruses, leading to SARS disease pandemics, and since 2019 to the rapid transmission and high mutation rate of SARS-CoV-2. In addition to several VOCs that have been circulating around the globe, recent studies have reported the identification of novel untracked SARS-CoV-2 lineages from unknown hosts. Given the well known relevance of the RBD–ACE2 interaction to initiating the entry of SARS-CoV-2 into host cells, a deep understanding of how different amino acids modulate the strength of the RBD–ACE2 interaction could contribute to dissecting the mechanisms underlying immune evasion, on the one hand, and could inform the design of a further therapeutic arsenal against novel SARS-causing species on the other hand.

In our search for potential substitutions that could lead to tighter binding, we found that RBD Q498Y yields the strongest predicted interaction. This result is consistent with previous *in silico* predictions that anticipated an increase in the binding affinity of RBD Q498Y to ACE2 (Li *et al.*, 2020; Starr *et al.*, 2020; Ahamad *et al.*, 2022).

To experimentally validate this prediction, we recombinantly expressed human ACE2, RBD wt and RBD Q498Y, and compared the kinetic profiles of binding using biolayer interferometry. Our binding studies thoroughly characterized the binding kinetics of Q498Y mutant RBD, providing $k_{on}$ and $k_{off}$ values and making a quantitative comparison with its wild-type and RBD $\beta$ variant counterparts possible. We found that RBD Q498Y has a binding affinity for ACE2 that is stronger than that of RBD wt. As a reference, the RBD $\beta$ variant, which carries three mutations in the residues located in the ACE2 binding site (Fig. 7), shows the strongest binding among the three RBD proteins tested. It is remarkable, however, that the single Q498Y replacement yields a 2.5-fold increase in the binding affinity, whereas the three mutations found in the RBD $\beta$ variant confer a fourfold increase. This result mirrors the impact of a single Q498Y mutation relative to a naturally occurring triple mutant SARS-CoV-2 VOC.

It is tempting therefore to speculate that Q498Y would contribute by tighter binding to the cell host, thus facilitating the subsequent infection process. Of course, the concomitancy of other mutations should be considered to analyze the impact on the overall binding to ACE2. Importantly, it should be noted that our binding studies were performed with monomeric RBD. Using monomeric RBD, we calculated a 2.5-fold stronger binding of the Q498Y mutant to ACE2 compared with its wild-type counterpart. Nevertheless, the SARS-CoV-2 spike protein has a trimeric structure, providing an avidity component that could further potentiate the effect of this mutation.

To confirm our results, we followed a different approach in which we assessed the ability of the three different RBD proteins to compete with surface-immobilized RBD wt for binding to ACE2 in solution. Again, RBD Q498Y showed a stronger interaction than that of RBD wt, while the RBD $\beta$ variant showed the tightest binding. We analyzed the individual impact of each substitution in the RBD $\beta$ variant. Our results are consistent with previous analyses performed by Han *et al.* (2021). In their study, they found that K417N reduces the binding to ACE2 from a $K_d$ of 22 n*M* (wild-type RBD) to a $K_d$ of 55 n*M* (RBD K417N), which may be associated with the abovementioned loss of salt bridges. A moderately increased affinity is observed for the E484K substitution ($K_d$ = 15 n*M*), while in agreement with the structural observations the N501Y substitution results in the largest impact ($K_d$ = 3.4 n*M*). Thus, in a similar manner to N501Y, the presence of a tyrosine residue at position 498 results in an increase in the overall binding affinity to ACE2.

The binding strengths of other SARS-CoV-2 variant RBDs have been analyzed by other groups. Using human ACE2 fused to a mouse IgG Fc domain, Han *et al.* (2021) determined the binding parameters of RBD from wild-type, alpha, beta, gamma, delta and omicron SARS-CoV-2 variants. The calculated binding strengths ($K_d$) followed the order alpha (5.4 n*M*) > gamma (11.0 n*M*) > beta (13.8 n*M*) > wild-type (24.6 n*M*) > delta (25.1 n*M*) > omicron (31.4 n*M*). Interestingly, the omicron variant, which has led to the highest infection rate in the population, is the variant with the weakest binding to ACE2. Therefore, even though all values fall in the low-nanomolar range and therefore represent high-affinity interactions, it cannot be concluded that stronger binding to ACE2 necessarily implies increased infection potential.

To dissect the molecular bases that underpin the enhanced binding conferred by Q498Y, we undertook structural studies and crystallized RBD Q498Y complexed with ACE2. The electron-density maps were of sufficient quality to trace the

**Figure 8**
Effect of temperature on the binding of RBD proteins to human ACE2. (*a*) Measurement of the binding affinity of RBD wt, RBD Q498Y and RBD $\beta$ at different temperatures using an ELISA-like plate assay (as described in Section 2). The graphs plot the absorbance at 450 nm (*y* axis) for each RBD concentration in n*M* (*x* axis; logarithmic scale). Each point represents a mean absorbance value ± standard deviation (SD). Data were fitted to a saturation, one-site specific binding model built into *GraphPad Prism* 9.0 to obtain the corresponding curve for each RBD and the $K_d$ values (Table 6). Assays were performed in triplicate. (*b*) Absorbance values for RBD wt, RBD Q498Y or RBD $\beta$ binding to ACE2 in a temperature-dependent manner.

RBD Q498Y–ACE2 complex. The overall docking mode is preserved from that of RBD wt (Shang *et al.*, 2020; Lan *et al.*, 2020), as expected from only a single amino-acid replacement. A closer inspection of the binding interface in the Q498Y complex reveals conservation of the polar network of interactions with Asp38 and Gln42 in ACE2, but a more populated nonpolar group of contacts. These are mediated by the bulky Tyr498 side chain that engages four residues in ACE2, which importantly are located in two different structural bodies of ACE2. Of these, Asp38, Tyr41 and Gln42 belong to the ACE2 N-terminal $\alpha$-helix, while Lys353 falls into a nearby loop connecting two strands of an antiparallel $\beta$-sheet. Interestingly, and consistent with previous computational predictions (Yi *et al.*, 2020), an additional $\pi$–$\pi$ stacking interaction established between Tyr498 of RBD and Tyr42 of ACE2 further contributes to the overall binding strength conferred by RBD Q498Y. In conclusion, it is conceivable that the larger number of contacts provided by the aromatic ring of Tyr498 constitutes the basis for the stronger adherence of RBD Q498Y. This observation is mirrored by a slower dissociation rate, as measured in our binding assays.

We also assessed the role of thermodynamics in the binding of RBD wt, RBD Q498Y and RBD $\beta$ to ACE2. In agreement with previous results by other groups (Prévost *et al.*, 2021), we observed that RBD–ACE2 binding is reduced at higher temperatures. This tendency is less accentuated in the RBD Q498Y mutant, yet decreased binding is still observed at 37°C. From a molecular point of view, these results suggest that at high temperatures the binding interfaces of RBD and ACE proteins have a reduced binding capacity, which could be a consequence of temperature-dependent protein plasticity and loss of an optimal conformation.

The Q498Y mutation has drawn the attention of several groups: for instance, in evaluation of its impact on the binding affinity to ACE2 using computational methods (Capponi *et al.*, 2021; Ahamad *et al.*, 2022; Li *et al.*, 2020). In *in vivo* assays, Leist and coworkers observed that its occurrence concomitant with Q493K and P499T is lethal in murine models of infection (Leist *et al.*, 2020). It must be noted that this triple mutant was examined *in vivo* with murine ACE2. In the crystal structures with human ACE2 Pro499 is not involved in direct contacts with the receptor, while Gln493 binds Lys31 and Glu35 via hydrogen bonds and His34 through van der Waals interactions. In murine ACE2, positions 31 and 34 are not conserved with regard to the human counterpart, being occupied by Asn and Gln residues, respectively. Since the residues surrounding Tyr498 are conserved across the human and murine species, it is tempting to speculate that the impact on the affinity provided by Tyr498 is also preserved in murine ACE2.

Furthermore, in a recent study by Smyth *et al.* (2022) the Q498Y mutation was detected in previously unidentified SARS-CoV-2 lineages found in wastewater samples from New York City (Smyth *et al.*, 2022). Whether these novel variants, which interestingly share mutations with the omicron VOC, arise from human or other animal sources remains to be investigated. Nevertheless, these findings indicate that the exposure of humankind to SARS-CoV-2 variants is higher than expected. As of 20 February 2022, five occurrences harboring the Q498Y mutation have been detected in humans in 8 180 793 sampled sequences (https://www.gisaid.org/). Thus, the actual prevalence of Q498Y is likely to be notably higher. Regarding its distribution, the first report was monitored in December 2021 in Argentina, while the most recent was registered in Germany in February 2022. Together, these data confirm the prevalence of and exposure to circulating and widespread SARS-CoV-2 variants and novel lineages carrying the Q498Y mutation.

In summary, we provide biophysical and X-ray structural data that describe the molecular mechanisms underlying the stronger affinity to ACE2 conferred by a substitution in SARS-CoV-2 RBD that has drawn the attention of the scientific community.

## Acknowledgements

JL-S is a Ramón y Cajal Investigator. We thank the staff of the XALOC beamline at ALBA Synchrotron for their assistance with X-ray diffraction data collection. We thank Maria Gilda Dichiara Rodriguez and Adela Rodriguez Fernández for their excellent technical support. Author contributions were as follows. Conceptualization, JL-S; methodology, JL-S, EEA, FG and JF-R; investigation, JL-S, EEA, FG and JF-R; funding acquisition, JL-S, FG and JF-R; supervision, JL-S; draft writing, JL-S, FG and EEA. The authors declare that they have no conflicts of interest. Funding for open access provided by Universidad Pública de Navarra (UPNA)-Conference of Rectors of Universities (CRUE).

## Funding information

This work was supported by Ramón y Cajal, Ministry of Science and Innovation, Government of Spain (grant RYC-2017-21683 to JL-S) and COVID 19 Ayudas la Investigación, Government of Navarre (Grant 0011-3597-2020-000010 to JL-S). This work was partially supported by grant No. PID2019-110167RB-I00/AEI/10.13039/501100011033 (to JFR). This work was partially funded by Israeli Science Foundation grant 2106/20 (to FG).

## Graphical abstract

The structural bases underpinning the higher affinity for the human receptor ACE2 conferred by a naturally occurring mutation (Q498Y) in the SARS‐CoV‐2 spike receptor‐binding domain are described.

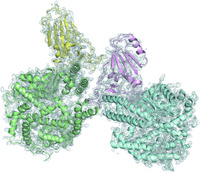

The full text for this article, hosted at journals.iucr.org, is unavailable due to technical difficulties.

## Supporting information

Supporting information for this article can be found here

