# Supplementary material for: Structural bases for the higher adherence to ACE2 conferred by the SARS-CoV-2 spike Q498Y substitution
Source: Acta Crystallogr D Struct Biol. 2022 Aug 25;78(Pt 9):1156–70. doi: 10.1107/S2059798322007677 (PMC9435600; doi:10.1107/S2059798322007677)
Supplement: Supplementary file 1 [file d-78-01156-sup1.pdf]

Acta Cryst D
STRUCTURAL
BIOLOGY

Volume 78 (2022)

Supporting information for article:

Structural bases for the higher adherence to ACE2 conferred by the SARS-CoV-2 spike Q498Y substitution

Elena Erausquin, Fabian Glaser, Juan Fernández-Recio and Jacinto Lopez-Sagaseta

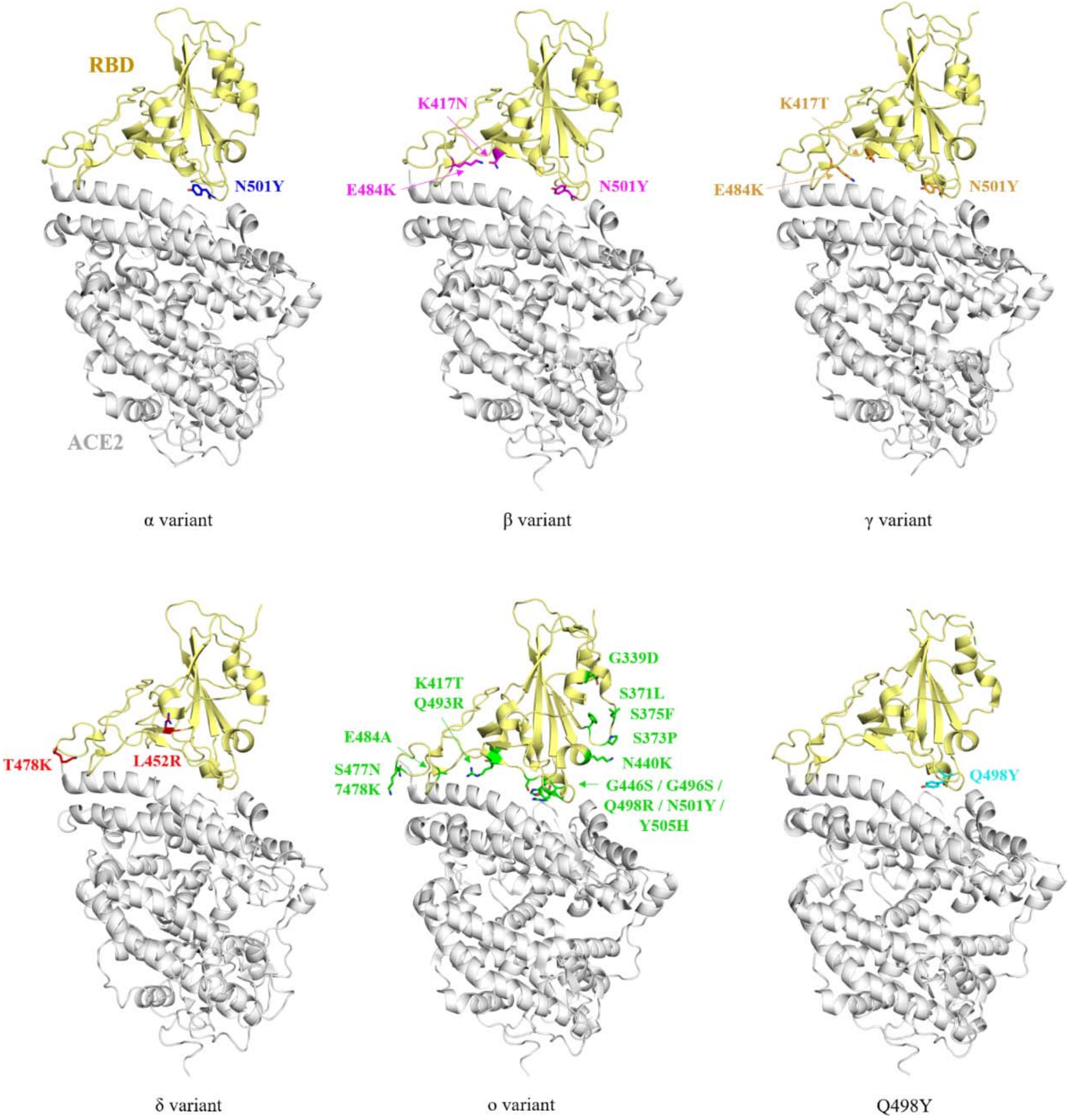

Figure S1 SARS-CoV-2 variants of concern and associated amino acid substitutions in the RBD. Cartoon representation of the RBD wt-ACE2 complexes (pale yellow and white colors, respectively) using the atomic coordinates deposited in the PDB under the accession number 6M0J. The amino acid substitution for each variant are highlighted in colors.

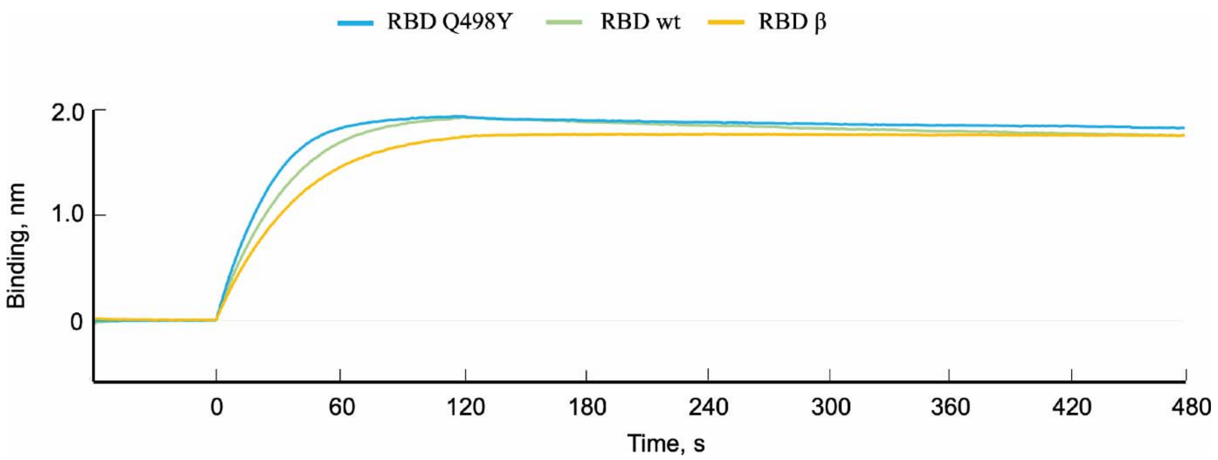

Figure S2 Comparative binding analysis of RBD wt, RBD Q498Y and RBD β to human ACE2. 200 nM of each RBD form was loaded on an ACE2-coated sensor and the association (120 s) and dissociation (360 s) curves were monitored for comparative purposes. Note that, according to our kinetics analyses (Table 1), Q498Y shows the highest k_{on} while RBD β has the slowest dissociation rate (k_{off}).

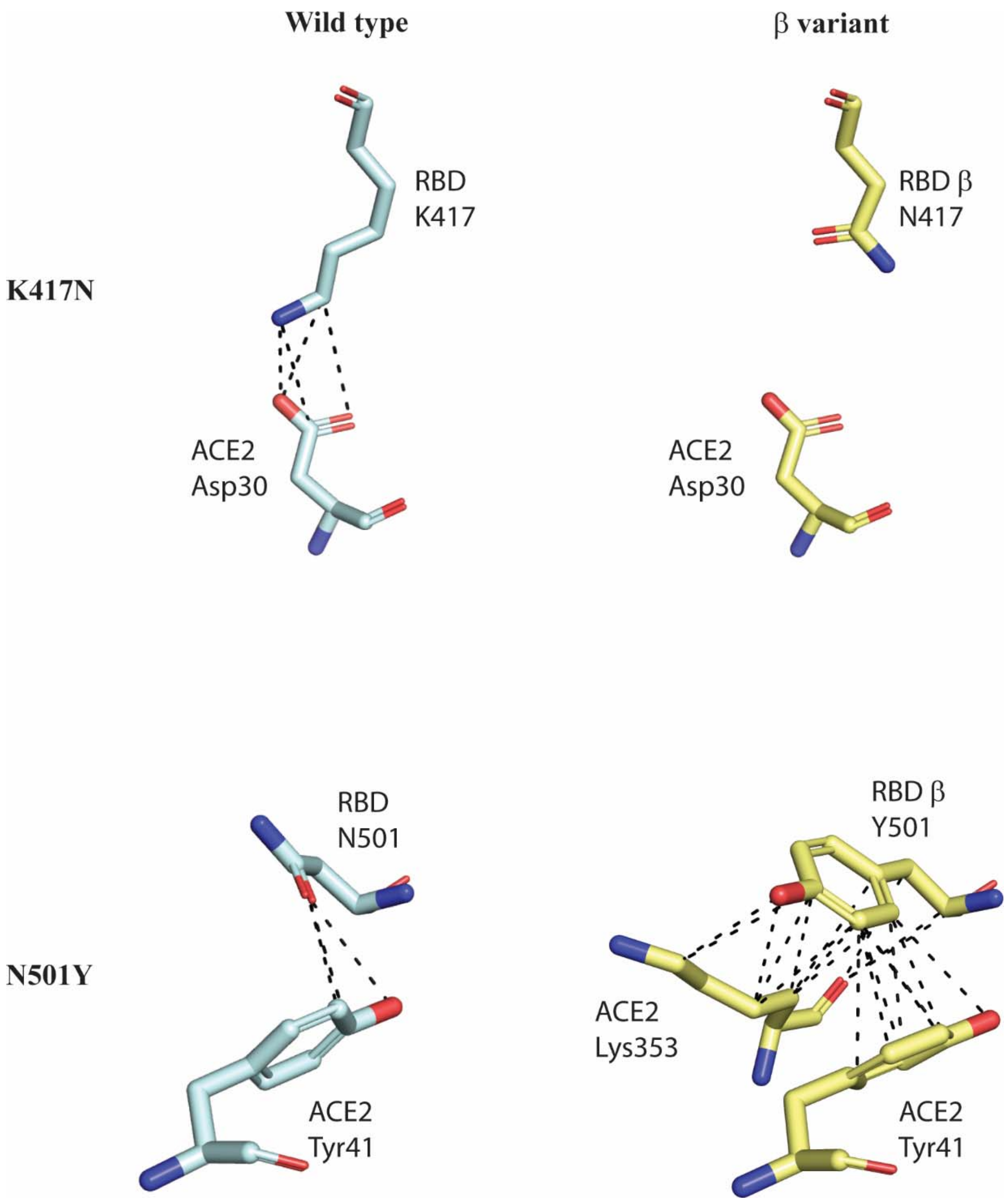

Figure S3 Structural analysis of the residues in RBD β VOC that differ from RBD wt and their role in binding to ACE2. The interactions established by the RBD WT K417 and N501 residues with ACE2 are shown represented as dashed lines (PDB ID 6M0J). Each residue is displayed in sticks format. RBD β 417N and Y501 are displayed on the right side for comparative purposes (PDB ID 7EKG).

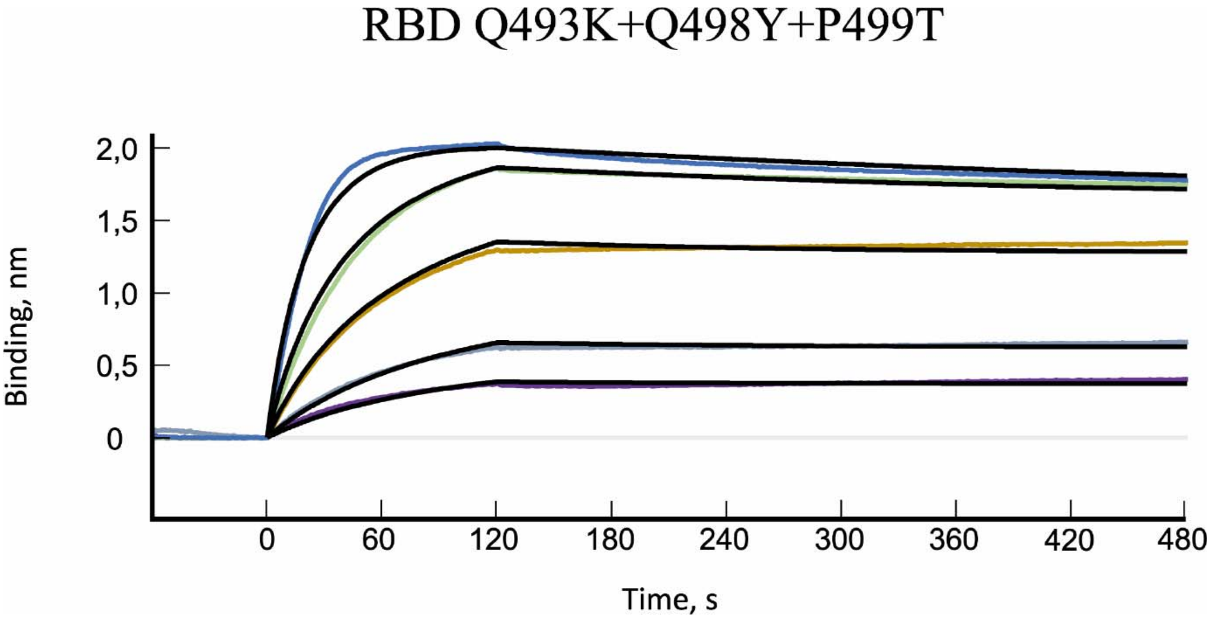

Figure S4 Binding kinetics of the triple mutant RBD Q493K/Q498Y/P499T to ACE2. ACE2 was captured through a 12xHisTag and exposed to increasing concentrations of the triple mutant. The association and dissociations traces were registered for the kinetic constants determined (see Table 1)
